# Recurrent Dissemination Despite Local Control in a Case of Hypervirulent Klebsiella pneumoniae Liver Abscess: A Case Report

**DOI:** 10.7759/cureus.85438

**Published:** 2025-06-05

**Authors:** Kohei Oka, Natsumi Yamamoto, Shiho Amano, Chiaki Sano, Ryuichi Ohta

**Affiliations:** 1 Community Care, Unnan City Hospital, Unnan, JPN; 2 Community Medicine Management, Faculty of Medicine, Shimane University, Izumo, JPN

**Keywords:** aged, bacterial, endocarditis, general medicine, hypervirulence, klebsiella pneumoniae, liver abscess, pyogenic, sepsis, spinal epidural abscess

## Abstract

An 82-year-old man with multiple comorbidities presented to a rural hospital with fever and reduced mobility and was diagnosed with a liver abscess due to hypervirulent *Klebsiella pneumoniae* (HVKP). Initial treatment with intravenous antibiotics led to radiological improvement, but the patient experienced recurrent bacteremia, low back pain, and eventually developed an epidural abscess, infectious endocarditis, and cerebral septic emboli. Despite antibiotic escalation and supportive care, his condition deteriorated, and surgical intervention was deemed unfeasible due to advanced sepsis and heart failure. He died on hospital day 27. This case highlights the diagnostic and therapeutic challenges of HVKP infections, especially in older adults with underlying illnesses. Even with early treatment and apparent control of the primary abscess, HVKP can disseminate hematogenously, leading to severe complications. Clinicians should maintain a high index of suspicion for systemic spread and consider prolonged treatment and closer monitoring, particularly in elderly or immunocompromised patients.

## Introduction

Hypervirulent *Klebsiella pneumoniae* (HVKP) is an emerging pathogen that differs from classical *K. pneumoniae* due to its high virulence and ability to cause severe infections in healthy individuals [1]. HVKP prevalence varies from 8.3% to 73.9%, depending on the region [2]. HVKP-associated liver abscesses have been predominantly reported in East Asia and have been recognized as a significant cause of invasive infections, often leading to metastatic complications such as endophthalmitis, meningitis, or pneumonia [3]. The mortality rate is from 3% to 31%, showing that prompt diagnosis and treatment are mandatory [1,3]. Unlike classical *K. pneumoniae*, HVKP strains exhibit hypermucoviscosity and possess distinct virulence factors, including capsular serotypes K1 and K2 and genes such as *rmpA* and *magA*, contributing to their pathogenicity [4].

HVKP-associated liver abscess can be effectively treated with prompt antibiotic treatment, but a high frequency of bacterial dissemination may complicate the cases [5]. Managing HVKP-associated liver abscess may need nuanced therapies, with the duration and doses of antibiotics adjusted. However, there is a lack of evidence for managing HVKP-associated liver abscesses [1]. This time, we experienced an older patient with HVKP-associated liver abscess initially effectively treated with antibiotics, eventually progressively worsening and causing peridural abscess and infectious endocarditis. Through this case report, we discuss the challenges of the HVKP-associated abscess and the realistic management of the disease in rural contexts.

## Case presentation

An 82-year-old Japanese man presented to our rural hospital with fever and impaired mobility. Three days before admission, he began experiencing vomiting and poor oral intake. On the day of presentation, he was found immobile in his room and transported to the hospital by ambulance. His medical history included type 2 diabetes mellitus (T2DM), dyslipidemia, hypertension, multiple cerebral infarctions, cerebral aneurysm, angina pectoris, hypothyroidism, chronic liver disease (CLD), and localized abdominal aortic dissection. His medications included azosemide of 30 mg, edoxaban of 30 mg, tolvaptan of 15 mg, dapagliflozin of 10 mg, and amlodipine of 5 mg daily. There was no history of recent international travel or known exposure to tuberculosis or other infectious diseases.

Upon arrival, his vital signs were as follows: body temperature, 38.5 °C; blood pressure, 143/82 mmHg; heart rate, 80 beats per minute; respiratory rate, 15 breaths per minute; and peripheral oxygen saturation (SpO₂), 97% on room air. He was alert and oriented. Physical examination revealed tenderness in the right upper quadrant of the abdomen and positive liver percussion tenderness. No other abnormal findings were noted.

Initial laboratory evaluation revealed leukocytosis with a white blood cell count of 10,600/μL (normal range: 3,500-9,100/μL), elevated liver enzymes, including aspartate aminotransferase (AST) 96 IU/L (normal: 8-38 IU/L), alanine aminotransferase (ALT) 61 IU/L (normal: 4-43 IU/L), alkaline phosphatase (ALP) 268 IU/L (normal: 40-130 IU/L IU/L), and gamma-glutamyl transpeptidase (γ-GTP) 243 IU/L (normal: 10-47 IU/L). Inflammatory markers were elevated with a C-reactive protein (CRP) level of 10.98 mg/dL (normal: <0.30 mg/dL) (Table 1).

**Table 1:** Initial laboratory data of the patient. CRP, C-reactive protein; eGFR, estimated glomerular filtration rate

Parameter	Level	Reference
White blood cells	10.6	3.5-9.1 × 10^3^/μL
Neutrophils	87.3	44.0-72.0%
Lymphocytes	4.7	18.0-59.0%
Hemoglobin	14.4	11.3-15.2 g/dL
Hematocrit	32.0	33.4-44.9%
Mean corpuscular volume	93.2	79.0-100.0 fL
Platelets	12.5	13.0-36.9 × 10^4^/μL
Total protein	7.4	6.5-8.3 g/dL
Albumin	3.3	3.8-5.3 g/dL
Total bilirubin	1.1	0.2-1.2 mg/dL
Serum glucose	153	70-126 mg/dL
Aspartate aminotransferase	96	8-38 IU/L
Alanine aminotransferase	61	4-43 IU/L
Lactate dehydrogenase	310	121-245 U/L
Blood urea nitrogen	22.7	8-20 mg/dL
Creatine kinase	277	56-244 U/L
Creatinine	1.00	0.40-1.10 mg/dL
eGFR	54.7	>60 mL/min/1.73 m^2^
Serum Na	140	135-150 mEq/L
Serum K	3.6	3.5-5.3 mEq/L
Serum Cl	101	98-110 mEq/L
CRP	10.98	<0.30 mg/dL
Urine test	-	-
Leukocyte	Negative	Negative
Protein	Negative	Negative
Blood	Negative	Negative

**Figure 1:**
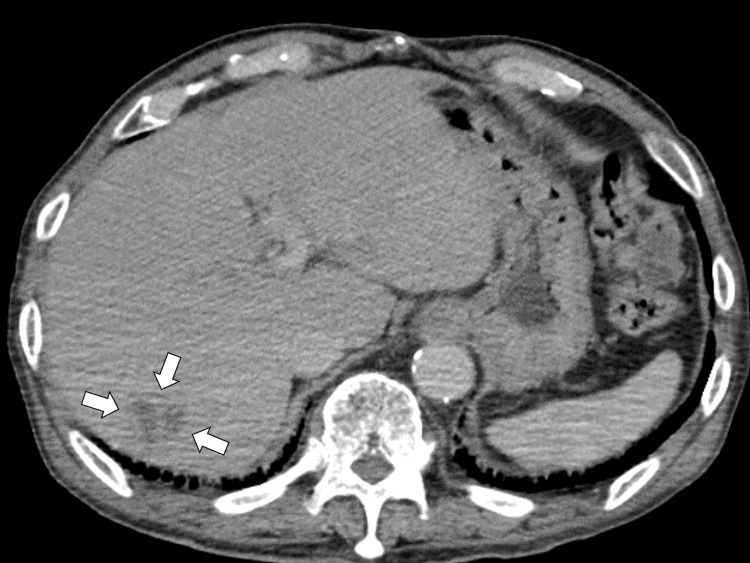
A dynamic contrast-enhanced abdominal computed tomography scan showing irregularly attenuated areas in the liver, consistent with hepatic abscesses (white arrows).

A plain computed tomography (CT) scan of the chest and abdomen showed bilateral pleural effusions, newly identified low-attenuation lesions in liver segments 5 and 7, and a previously known compression fracture at the T12 vertebra. A dynamic contrast-enhanced abdominal CT confirmed ring enhancement around the liver lesions, consistent with hepatic abscesses measuring approximately 2.2 cm in segment 5 and 2.5 cm in segment 7, without any enlargement of the previous aortic dissection (Figure 1).

**Figure 2:**
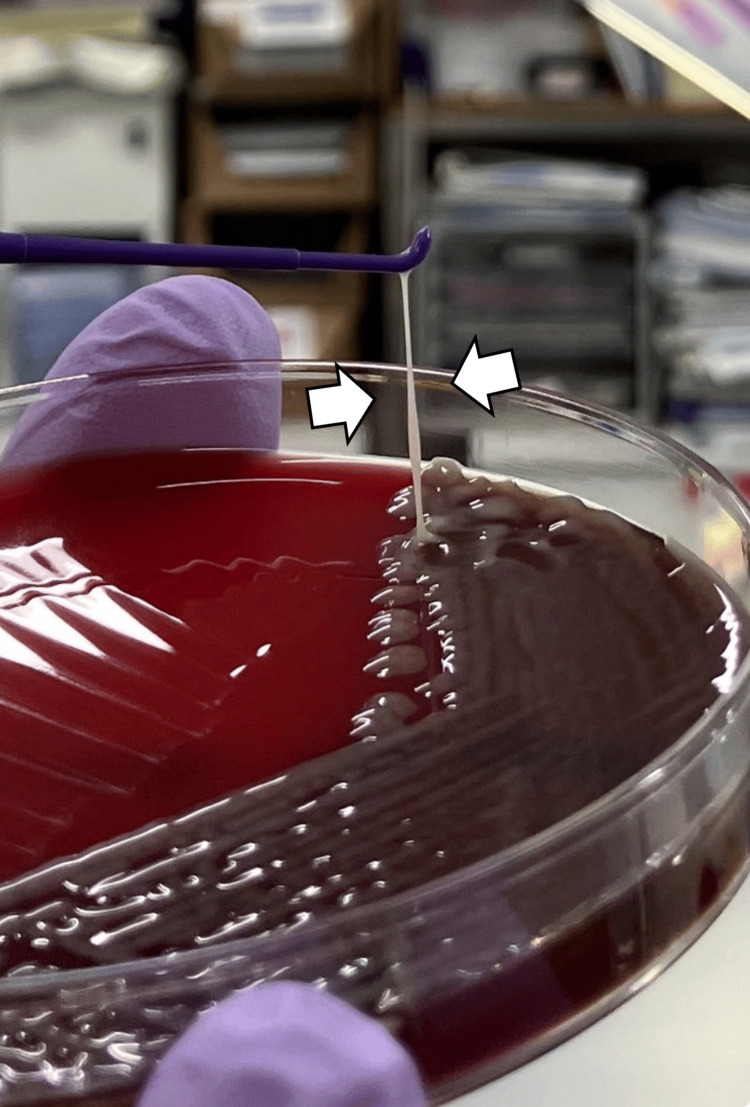
Positive string test demonstrating the hypermucoviscous phenotype, indicative of hypervirulent Klebsiella pneumoniae (HVKP) (white arrows).

Based on the diagnosis of a liver abscess, a consultation was made with the surgical team regarding percutaneous drainage. Still, drainage was not indicated at that time, given the abscess size of approximately 2 cm in diameter. Empirical antimicrobial therapy with intravenous cefmetazole (4 g/day) was initiated. On hospital day 5, blood cultures grew *Klebsiella pneumoniae* sensitive to ampicillin/clavulanate, and a positive string test confirmed the hypermucoviscous phenotype, indicating HVKP (Figure 2).

**Figure 3:**
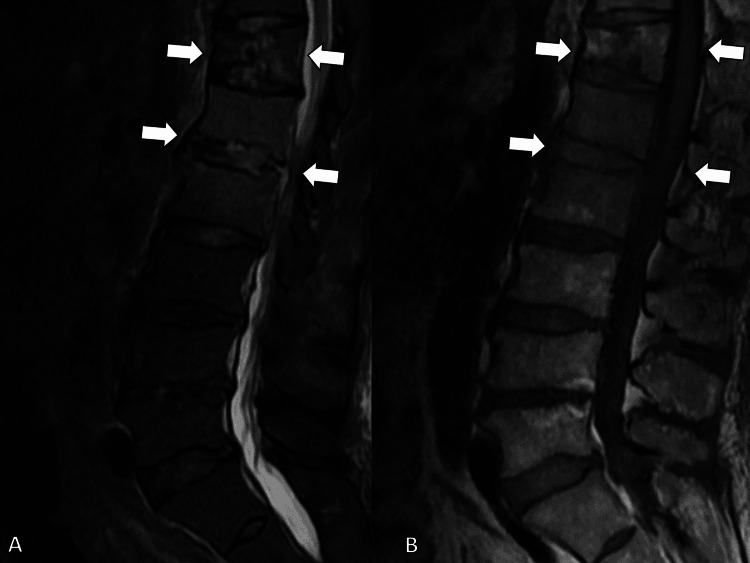
Magnetic resonance imaging of the lumbar spine revealing signal changes at the L1/L2 level (white arrows). (A) Short τ inversion recovery image, and (B) T1-attenuated image.

On hospital day 6, due to progressive low back pain, magnetic resonance imaging (MRI) of the lumbar spine was performed, revealing signal changes at the L1/L2 level, suggestive of vertebral osteomyelitis and/or discitis (Figure 3).

The orthopedic team was consulted and considered the possibility of a compression fracture and pyogenic spondylitis. Concurrent liver ultrasound showed reduced abscess size, so cefmetazole therapy was continued.

By hospital day 13, the patient had become afebrile, and his antimicrobial therapy was switched to oral amoxicillin/clavulanate (500/125 mg three times daily) and metronidazole (500 mg three times daily). However, on hospital day 14, he developed chills and recurrent fever up to 39.1 °C. Blood cultures were reobtained, and intravenous ampicillin/sulbactam (ABPC/SBT) of 12 g/day was initiated. The fever was resolved the following day, but Gram-negative rods were detected in blood cultures and later confirmed as *Klebsiella pneumoniae* sensitive to ampicillin/clavulanate.

**Figure 4:**
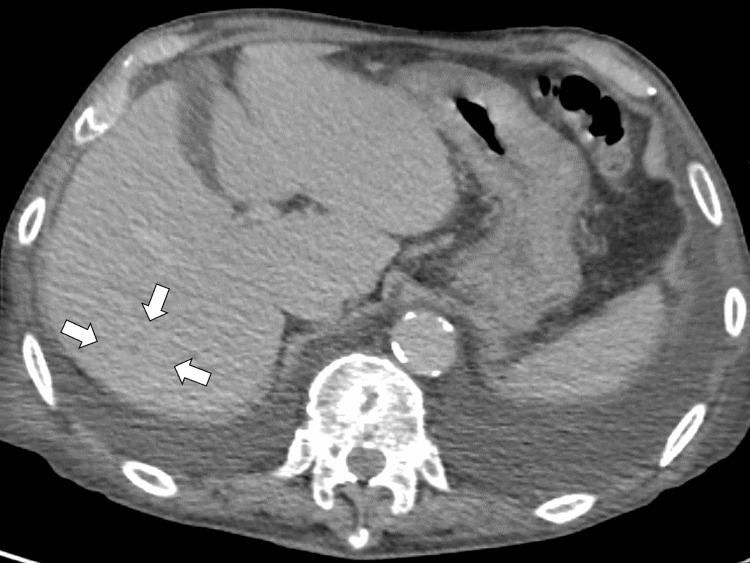
Repeat contrast-enhanced computed tomography showing further reduction of the hepatic abscess and no new infectious foci (white arrows).

On hospital day 17, the patient developed increased oxygen requirements, poor oral intake, and low-grade fever up to 37.8 °C. Transthoracic echocardiography (TTE) revealed that no definitive vegetations were identified. On hospital day 18, the patient developed hemoptysis and recurrent fever of approximately 38.5 °C. Repeat contrast-enhanced CT showed further reduction of the hepatic abscess and no new infectious foci (Figure 4).

Non-invasive positive pressure ventilation (NPPV) was commenced for respiratory support. On hospital day 19, the respiratory status temporarily improved, and hemoptysis subsided. Blood cultures drawn the previous day once again yielded *Klebsiella pneumoniae*.

**Figure 5:**
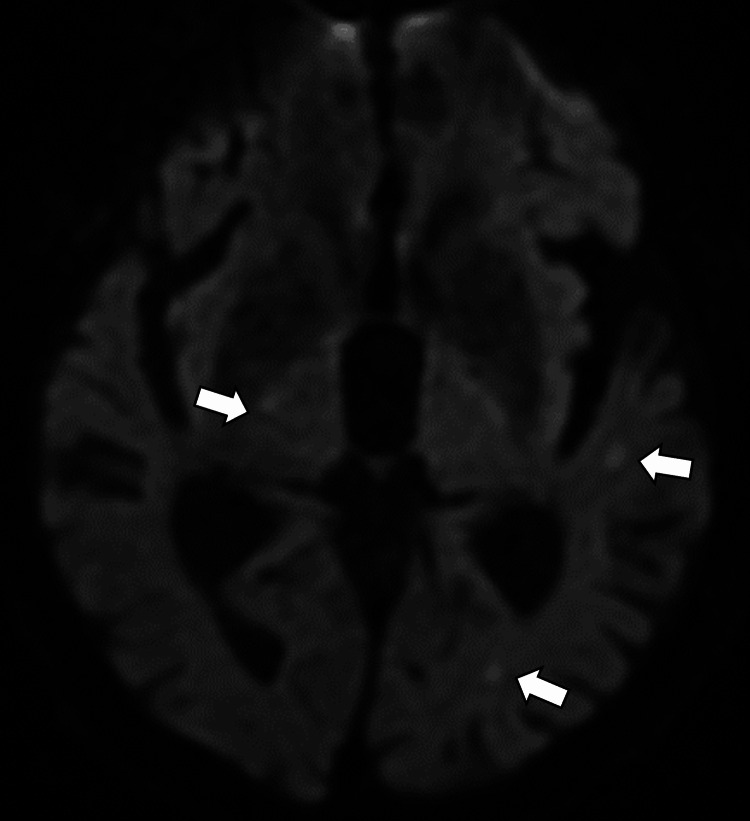
Brain magnetic resonance image revealing multiple hyperintense lesions on diffusion-weighted imaging (white arrows).

On hospital day 20, the patient developed altered consciousness. Brain MRI revealed multiple hyperintense lesions on diffusion-weighted imaging (DWI) and hypointense lesions on apparent diffusion coefficient (ADC) mapping, suggestive of septic emboli (Figure 5).

**Figure 6:**
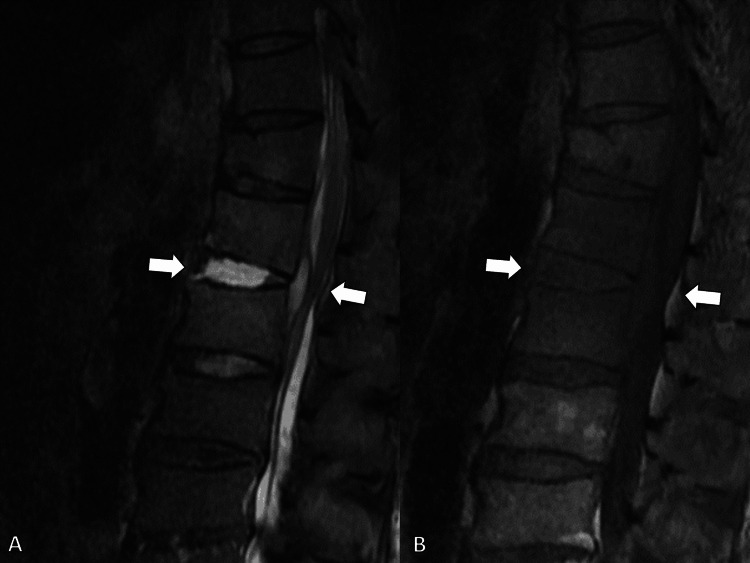
Magnetic resonance imaging of the lumbar spine showing dorsal epidural signal changes at L1/L2, consistent with an epidural abscess (white arrows). (A) Short τ inversion recovery image, and (B) T1-attenuated image.

MRI of the lumbar spine revealed dorsal epidural signal changes at L1/L2, consistent with an epidural abscess (Figure 6).

Cerebrospinal fluid (CSF) analysis revealed a mildly elevated white cell count (12/μL) and markedly elevated protein (465 mg/dL), indicating involvement of the central nervous system.

The patient was transferred to a tertiary care hospital for possible epidural drainage and advanced management. However, due to his deteriorating condition from sepsis and congestive heart failure (CHF), surgical intervention was considered unfeasible. Despite continued antimicrobial and supportive therapy, the patient died on hospital day 27 from sepsis and heart failure.

## Discussion

This case illustrates the clinical challenges posed by HVKP-associated liver abscesses in older adults, particularly concerning disease progression and treatment strategies. HVKP is increasingly recognized for its ability to cause invasive infections, including liver abscess, endophthalmitis, meningitis, and pneumonia, even in immunocompetent hosts [2,6]. However, elderly patients with comorbidities may present atypically and are more vulnerable to persistent or relapsing infections.

Although initial imaging demonstrated regression of the liver abscess after antimicrobial therapy, our patient developed severe metastatic complications, including vertebral osteomyelitis and infectious endocarditis. Previous studies have emphasized that HVKP is capable of hematogenous dissemination despite adequate treatment of the primary infection site [7]. This phenomenon is partly attributable to its enhanced virulence factors, particularly the K1/K2 capsular types and rmpA/magA genes, contributing to hypermucoviscosity, immune evasion, and systemic spread [8]. In our case, the string test confirmed the hypermucoviscous phenotype of the isolated strain, supporting the diagnosis of HVKP.

Importantly, although HVKP liver abscesses are often described as presenting with abrupt onset and high-grade fever, our patient followed a more indolent course, highlighting the diagnostic and management challenges in the elderly [9]. Older adults often demonstrate atypical signs of infection, and immunosenescence may blunt systemic responses, delaying recognition of dissemination [10]. Furthermore, the presence of comorbidities such as diabetes mellitus and chronic liver disease, both known risk factors for HVKP infection, likely contributed to this protracted disease course [11]. In particular, diabetes mellitus impairs innate immune functions, such as neutrophil chemotaxis, phagocytosis, and microbial killing, thereby predisposing patients to invasive infections [11]. Moreover, hyperglycemia promotes bacterial growth and provides a favorable environment for HVKP colonization and dissemination [11].

While early and aggressive antibiotic therapy is the standard of care, there is no consensus on the optimal duration or intensity of treatment for HVKP-associated infections [12]. Some retrospective studies suggest prolonged antibiotic courses and early percutaneous drainage improve outcomes, but such interventions may be limited in rural or resource-constrained settings [13-15]. In our case, drainage was not initially indicated by surgical consultation, which may have contributed to incomplete bacterial eradication and subsequent systemic spread.

The string test, a simple phenotypic method, can aid in the early presumptive identification of hvKp strains. The test is considered positive when a viscous string of >5 mm long is formed by stretching bacterial colonies on an agar plate using a bacteriological loop or needle [13,14]. This *hypermucoid* phenotype is associated with enhanced virulence and invasiveness, and a positive string test result may prompt clinicians to consider more aggressive diagnostic and therapeutic approaches [12].

Regarding antimicrobial therapy, hvKp strains are typically susceptible to a broad range of antibiotics; however, empiric treatment should be guided by local resistance patterns and adjusted based on susceptibility results [12]. Third-generation cephalosporins such as ceftriaxone are commonly used initially [12]. In cases with severe or disseminated disease, combination therapy with extended-spectrum β-lactams (e.g., piperacillin-tazobactam) or carbapenems may be considered, especially in settings concerned with multidrug resistance or ESBL-producing strains [8]. Treatment duration is generally extended (often 4-6 weeks), particularly when deep-seated abscesses or complications such as osteomyelitis are present [3].

This case also underscores the limitations of relying solely on radiological improvement of the primary abscess to guide treatment decisions. Even in improved imaging findings, subclinical bacteremia and metastatic foci may persist, warranting more comprehensive follow-up strategies, including serial blood cultures, inflammatory markers, and possibly whole-body imaging in high-risk cases.

## Conclusions

HVKP liver abscess in elderly patients can follow a slow but progressive clinical course, despite appropriate initial therapy. Clinical vigilance should remain high for delayed complications, even when the primary lesion appears to be resolving. Tailored treatment strategies, emphasizing prolonged antibiotic use, multidisciplinary evaluation, and possibly earlier intervention, may be necessary, particularly in older or immunocompromised populations. Future prospective studies are needed to establish evidence-based guidelines for HVKP management, especially in aging societies where such infections may become increasingly common.
